# Medication Adherence Mediates the Association between Type D Personality and High HbA1c Level in Chinese Patients with Type 2 Diabetes Mellitus: A Six-Month Follow-Up Study

**DOI:** 10.1155/2017/7589184

**Published:** 2017-02-09

**Authors:** Xuemei Li, Min Gao, Shengfa Zhang, Huiwen Xu, Huixuan Zhou, Xiaohua Wang, Zhiyong Qu, Jing Guo, Weijun Zhang, Donghua Tian

**Affiliations:** ^1^School of Social Development and Public Policy, China Institute of Health, Beijing Normal University, Beijing 100875, China; ^2^Clinics of Cadre, Department of Outpatient, General Hospital of the People's Liberation Army (301 Hospital), Beijing 100853, China; ^3^Department of Public Health Sciences, University of Rochester School of Medicine & Dentistry, Rochester, NY 14642, USA; ^4^Department of Sociology, Huazhong University of Science and Technology, Wuhan, Hubei 430074, China

## Abstract

*Aims*. To examine the association between Type D personality and HbA1c level and to explore the mediating role of medication adherence between them in patients with type 2 diabetes mellitus (T2DM).* Methods*. 330 patients went on to complete a self-report measure of medication adherence and the HbA1c tests. Chi-square test, *T* test, Ordinary Least Square Regression (OLS), and Recentered Influence Function Regression (RIF) were employed.* Results*. Patients with Type D personality had significantly higher HbA1c value (*P* < 0.01). When Type D personality was operationalized as a categorical variable, SI was associated with HbA1c (*P* < 0.01). When NA, SI, and their interaction term were entered into regression, all of them were no longer associated with HbA1c level (*P* > 0.1). On the other hand, when Type D personality was operationalized as a continuous variable, only SI trait was associated with HbA1c level (*P* < 0.01). When NA, SI, and NA × SI term together were entered into regression, only SI was not related to HbA1c level. Furthermore, medication adherence had a significant mediation effect between Type D personality and HbA1c, accounting for 54.43% of the total effect.* Conclusion*. Type D personality was associated with HbA1c in direct and indirect ways, and medication adherence acted as a mediator role.

## 1. Introduction

Adherence has been defined by WHO as “the extent to which a person's behavior-taking medications, following a diet, and/or executing lifestyle changes, corresponds with agreed recommendations from a health care provider” [1]. Adequate adherence enhances treatment safety and treatment effectiveness and further leads to reduced mortality, morbidity, and considerable direct and indirect costs to healthcare system [2, 3]. In addition, accumulating evidence presented that Type D personality is significantly associated with medication adherence in patients with chronic disease [4, 5] and has a deleterious influence on the patient's self-behavior [6]. Patients with Type D personality may be nonadherent because of the concerns about negative side-effects of medication and the inability to talk about these concerns [7], or due to fewer health-enhancing behaviors, such as eating sensibly and getting a regular medical examination [6]. Additionally, people with high SI might adhere to treatment less because they tend to use more avoidant and passive coping strategies (e.g., denial) when dealing with (medical) problems [8]. Previous studies revealed that poor self-management such as medication adherence can lead to life-threating complications including microvascular or macrovascular disease for the patients with T2DM [9], or with chronic heart failure [10], and/or with peripheral arterial disease [11].

Diabetes is a main contributor for death, disability, and rising medical expenses in both developed countries and developing countries. The majority of diabetes-related complications may be the result of higher blood glucose level, especially the elevated blood sugar over a prolonged period of time [12]. Glycated haemoglobin (HbA1c), which reflects the average blood glucose level over 8–12 weeks and provides a useful longer-term gauge of blood glucose control [13], has been used as an objective marker of average glycaemic control for a long time [14]. Also, HbA1c has been endorsed as a diagnostic test for diabetes by the World Health Organization (WHO), the International Diabetes Federation (IDF), and the American Diabetes Association (ADA) [15–18].

Recently, a six-month follow-up study, conducted by our research team, showed that Type D personality predicted poor medication adherence in Chinese patients with Type 2 diabetes mellitus (T2DM) (*P* < 0.001) [19]. A rapidly growing number of studies have also suggested that poor medication adherence will lead to higher blood glucose level and increased risks of complications. For example, a more recent study, conducted in Maastricht, indicated that people with Type D personality had significantly higher levels of HbA1c and fasting glucose (*P* = 0.008) [20]. Therefore, this study aimed to explore whether there is a direct association between Type D personality and higher HbA1c level, or whether medication adherence acted as a mediating role, or whether both the direct and the indirect association coexist between Type D personality and HbA1c in Chinese population with T2DM, after controlling for demographic, clinical, and behavioral characteristics.

## 2. Materials and Methods 

### 2.1. Participants

The sample procedures have been introduced in our previously published articles [19, 21]. The baseline survey was conducted from March l to May 31, 2012; patients completed measures of Type D personality and provided demographic information, clinical outcomes, and so on. Six months later, patients were contacted again to complete a self-report measure of medication adherence and HbA1c test. Eventually, 330 participants completed the follow-up investigation and accepted HbA1c test after receiving informed consent, at the six-month follow-up (Time 2, T2) [19]. No significant differences were found between the respondents and nonrespondents in gender, HbA1c level at baseline, and Type D personality, except for the age [19].

### 2.2. Measurements

*Demographic Information and Clinical Characteristics*. The variable information at baseline and six months later in this study has been described in our previous articles [19, 22]. Specifically, the demographic and socioeconomic factors, life behavior factors, and clinical characteristics information were collected at baseline. Meanwhile, medication adherence information was also gathered at six months later. 

*Type D Personality*. Type D personality was assessed by using the Chinese version of DS14 scale [23], consisting of Negative Affectivity (NA) and Social Inhibition (SI). The cut-off of ≥10 on both subscales indicating Type D personality. In this study, Cronbach's *α* were 0.89 and 0.93 for the NA and SI [19], respectively. 

*HbA1c Value*. In this study, HbA1c, which was obtained at baseline and six months later, is used as the marker of average glycaemic control. It has been reported that there is a strong positive correlation between HbA1c and blood glucose [17, 18]. The results of Diabetes Control and Complications Trial (DCCT) showed that when HbA1c increases 1%, the average blood glucose increases by 1.95 mmoL/L [24]. 

*Medication Adherence*. The Chinese version of the Medication Adherence Scale (MMAS-8-CN) was adopted to evaluate medication adherence [25]. In this study, Cronbach's *α* based on standardized items is 0.84 [19].

### 2.3. Statistical Analyses

In order to explore the relationship between Type D personality and HbA1c value in patients with T2DM, the following analytic strategies were employed. Firstly, since the dependent variable HbA1c was a continuous variable, Ordinary Least Square (OLS) regression was employed to explore the relation of the Type D personality and HbA1c value. Secondly, the method of Recentered Influence Function Regression (RIF) was employed to estimate directly the impact of the explanatory variables on the distributional statistic of interest. RIF showed how to formally decompose changes in the distribution of the dependent variable (HbA1c) into the contribution of independent factors (Type D personality). The dependent variable (HbA1c) was divided into several parts by quantile: 25th, 50th, 75th, in order to explore how Type D personality influence HbA1c value [26]. Thirdly, Type D personality was regarded as a classified variable; that is, it was recoded into a binary variable based on whether both NA and SI scored above the recommended cut-off (>10); then the interaction term of NA × SI was entered to test whether it could explain additional variance. Fourthly, NA and SI were treated as continuous variables; in addition, the multiplicative NA × SI term was also entered. Finally, Product of Coefficients Approach was employed to identify whether medication adherence is an intervening variable between Type D personality and HbA1c [27].

### 2.4. Ethics Statement

This study was approved by the Ethics Committee of School of Social Development and Public Policy at Beijing Normal University and the Ethics Committee of the First Affiliated Hospital of the General Hospital of PLA. All patients provided written informed consent, and all personal information was kept confidential and reporting was made anonymous.

### 2.5. Availability of Supporting Data

The database set was available for all authors of the study and will be available for other noncommercial researchers on request.

## 3. Results

### 3.1. Subjects Characteristics

Subjects' characteristics have been described in our previous article [19]. Among 330 participants, the average age was 57.23 years old, and 158 (47.88%) were males. In addition, there was a significant difference in the reimbursement ratio of treatment costs between T2DM patients with or without Type D personality (*P* = 0.016) (Table 1). However, there was no significant difference in personal income last year between T2DM patients with or without Type D personality (*P* = 0.368) (Table 1).

### 3.2. Clinical Characteristics

As shown in Table 2, at baseline, patients with Type D personality presented longer duration (*t* = −2.82, *P* = 0.005), more complications (*t* = −1.85, *P* = 0.065), and higher BMI scores (*t* = −2.37, *P* = 0.018), compared with that without Type D personality. However, HbA1c level (*t* = 0.55, *P* = 0.581) and cost of treating diabetes (*t* = −1.58, *P* = 0.114) were not significantly different between the T2DM patients with or without Type D personality. At the followup, the average of HbA1c level was 7.02 (SD = 1.003), with the range of 5.2–14.2. Patients with Type D personality presented higher HbA1c value (*t* = −5.88, *P* < 0.001) and lower medication adherence (*t* = 2.257, *P* < 0.001), compared with their counterparts.

### 3.3. Type D Personality Predicted Higher HbA1c Level at T2

As shown in Table 3, the first step of Ordinary Least Square Regression (OLS) included age, gender, and education variables, which did not account for a significant amount of HbA1c at T2, and the total adjust-*R*^2^ was 0.092. The clinical factors and behavior factors explained an additional 74.5% of the variance in step 2. There is one point which needs attention; that is, Type D personality had a significant influence on higher HbA1c level (*β* = 0.357, *P* < 0.01), which explained the additional 0.7% of the variance, in the final step.

As shown in the fourth to sixth columns of Table 3, Recentered Influence Function Regression (RIF) further confirmed the effect of Type D personality on HbA1c level at T2. Type D personality had a significant association with high (75th) percentile of HbA1c value (*β* = 0.646, *P* < 0.01), but it was not related to low (25th) percentile and median (50th) percentile of HbA1c value. Concurrently, the covariates such as age (*β* = 0.020, *P* < 0.05) and marital status (*β* = 0.252, *P* < 0.01) were also associated with high percentile of HbA1c significantly. Meanwhile, clinical characteristics also influenced HbA1c significantly; the subjects with long duration (*β* = −0.077, *P* < 0.01) tended to have lower HbA1c level; however, those with more complications (*β* = 0.161, *P* < 0.01) and higher medical cost (*β* = 0.069, *P* < 0.05) tended to have higher HbA1c level among patients with medium or above HbA1c level. Interestingly, the subjects with diabetes family history were inclined to have lower HbA1c level (*β* = 0.506, *P* < 0.01). In terms of behavior factors, sleep time per day had a significantly positive association with HbA1c level among patients with high HbA1c (*β* = 0.239, *P* < 0.01). Among the patients with low HbA1c percentile, however, exercise time per week (*β* = 0.096, *P* < 0.01) and drinking alcohol (*β* = −0.310, *P* < 0.05) were significantly associated to HbA1c level.

As shown in Table 4, Type D personality was operationalized as a categorical variable [28], that is, NA and SI, which were regarded as the categorical variables, were entered into the regression model (step 1). There was a different influence on HbA1c level at T2; SI was significantly associated with HbA1c (*β* = 0.395, *P* < 0.01). However, when NA, SI, and their interaction term (NA × SI term) were entered into multiple regression model, all of them were no longer significantly associated with HbA1c level (*P* > 0.1), and total adjust-*R*^2^ remained unchanged (step 2).

To further explore the associations between NA/SI trait and HbA1c level at T2, NA and SI, which were regarded as continuous variables, were entered into multiple regression model (step 3). The results showed that SI trait has a significantly positive correlation with HbA1c level at T2 (*β* = 0.034, *P* < 0.01), but NA trait was not associated with HbA1c level at T2 significantly (*P* > 0.1). One thing to be noted, when NA, SI, and NA × SI term together were entered into the Multiple Regression (step 4), NA (*β* = −0.031, *P* < 0.05) and their interaction term (*β* = 0.004, *P* < 0.05) were related to HbA1c level at T2.

### 3.4. Medication Adherence Acted as a Mediator Variable

**Figure 1:**
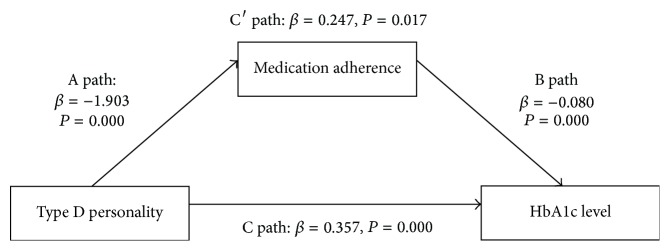
Medication adherence as a mediator between Type D personality and Type D personality after discharge. Note: A path: the independent variable (Type D personality) significantly influences the dependent variable (HbA1c level at T2) in the absence of the mediator (medication adherence). B path: the mediator (medication adherence) has a significant unique effect on the dependent variable (HbA1c level at T2) and also means that the direct effect of medication adherence on the dependent variable (HbA1c level at T2). C path: the independent variable (Type D personality) significantly influences the dependent variable (HbA1c level at T2). C′ path: the independent variable (Type D personality) significantly influences the dependent variable (HbA1c level at T2) on the addition of the mediator (medication adherence).

As shown in Table 5 and Figure 1, Type D personality had a direct and indirect influence on HbA1c level at T2 in patients with T2DM. Based on the results above, a hypothesis was proposed that medication adherence might be the mediator variable between Type D personality and HbA1c level at T2. Therefore, Product of Coefficients Approach was employed to estimate indirect effects of Type D personality through medication adherence in a simple mediation model (Figure 1): (1) A path represents the direct effect of Type D personality on medication adherence (*β* = −1.903, *P* < 0.01); (2) B path represents the direct effect of medication adherence on HbA1c level at T2 (*β* = −0.080, *P* < 0.01); (3) C path represents the direct effect of Type D personality on HbA1c level at T2 (*β* = 0.357, *P* < 0.01); (4) C′ path presents the coeffects of Type D personality and medication compliance on HbA1c level at T2 (*β* = 0.247, *P* < 0.05). The results confirmed the hypothesized mediating role for medication adherence between Type D personality and HbA1c level at T2. Based on the results of Product of Coefficients Approach, medication adherence had a significant mediation effect between Type D personality and HbA1c level at T2, and the coefficient is 42.64%, and accounting for 54.43% of the total effect (Table 5).

## 4. Discussion

To the best of our knowledge, this may be the first study to explore the potentially important mediational pathway between Type D personality and HbA1c level in patients with T2DM.

In this study, we have identified that Type D personality is an independent predictor of HbA1c level at T2, after controlling demographic, clinical and behavior factors (*β* = 0.357, *P* < 0.01), especially for patients with high (75th) percentile of HbA1c value. In addition, the constituent components of Type D personality (NA and SI) had different influence on HbA1c. As a categorical or continuous variable, SI was significantly associated with HbA1c, whereas NA was not. But when NA, SI, and their interaction term (NA × SI term) together were entered into the regression as categorical variables, all of them were no longer significantly associated with HbA1c (*P* > 0.1). However, when NA, SI, and their interaction term (NA × SI term) together were entered into the regression as continuous variables, NA and the interaction term (NA × SI term) had significant associations with HbA1c level at T2. In addition, the study also suggested that medication adherence was an important mediational pathway between Type D personality and HbA1c level at T2 in patients with T2DM, and the coefficient is 42.64%.

The findings showed that Type D personality was significantly associated with higher HbA1c level at T2 in patients with T2DM, which were similar to the high prevalence rates of Type D personality in other patient groups such as those suffering from hypertension, myocardial infarction, and heart failure [4, 29]. Personality is formed by family environment, school education, personal physical condition, and social environment and other factors. Several studies indicated that Type D personality predicted the lower objective assessed adherence in patients with obstructive sleep apnea syndrome [30] and was strongly associated with lower adherence to oral appliance therapy in patients with sleep-disordered breathing [31] and displayed inadequate self-management in patients with heart failure [32]. Another study showed that people with Type D personality are more inclined to be worried, anxious, pessimistic, and angry and inhibit self-expression in social interactions in order to avoid disapproval or rejection by others [33]. This study also showed that T2DM patients with Type D personality have poorer medication adherence and have more difficulty in controlling HbA1c level, compared with that without Type D personality. Furthermore, Type D personality has a strong influence on high percentile rank of HbA1c level at T2, which means, for patients with higher HbA1c, the effect of Type D personality is becoming more obvious; meanwhile, Type D personality also has a weak influence on low percentile rank of HbA1c.

To explore the mediating effect of medication adherence between Type D personality and HbA1c level was another important contribution of this study. It is well known that the issues regarding the relationship between Type D personality and health outcomes remained unclear. Although, recent studies found that Type D personality may be related to immune activation [34], hyperactivity of the hypothalamic-pituitary-adrenal axis [35], greater cardiovascular reactivity to stress [36], engagement in fewer health-related behaviors [6], depression [37], and suboptimal consultation behaviors [38]. Furthermore, there was also some evidence for a number of potential psychobiological mechanisms that may operate between Type D personality and poor health outcomes [35, 36, 39] and increasing evidence for behavioral mechanisms including self-management behaviors in cardiac patients [6, 40]. It is easy to understand that medication adherence, as a typical self-management behavior, should have a significant effect on HbA1c level. Once patients do not adhere to medication, the blood glucose level of patients will inevitably be influenced by the poor medication adherence. Previous studies have identified that high level of HbA1c will increase risks of having relevant complications [28, 41]. Therefore, after controlling for demographic, clinical, and behavioral characteristics, the questions on whether there is a direct association between Type D personality and HbA1c level, or whether medication adherence acts as a mediating role, or whether both the direct and the indirect association coexist between the Type D personality and HbA1c level were especially important and should be further resolved.

In this study, the results of Product of Coefficients Approach (PCA) analysis showed that medication adherence played a significant mediating effect between Type D personality and HbA1c level, which means Type D personality not only had a direct but also had an indirect influence on HbA1c level through medication adherence. In other words, as a negative personality, Type D personality may directly influence the HbA1c level through affecting patients' enthusiasm or activating a certain inherent mechanism in vivo, which should be further explored by conducting interdisciplinary research in the future; concurrently, it also has an adversely influence on the HbA1c level through lowering patients' medication adherence. Naturally, in this way, diabetic condition should certainly become more difficult to be controlled, and HbA1c level would remain obstinately high.

Traditionally, Type D personality was always employed as a categorical variable based on the scores of NA and SI above the median split [42]; however, the issue, which should not be ignored, considering Type D personality as a dichotomized variable may lead to a wrong outcome [43]. Therefore, the question on what Type D personality should be regarded as a continuous variable or a categorical variable still remains unclear. A recent study also suggested that Type D personality may be better considered as a dimensional construct than a categorical construct, because the whole range of data can more objectively reveal the facts and details [44]. However, a 5-year follow-up of 541 patients with CAD showed that continuous (NA × SI interaction) and dichotomized measures of Type D were associated with cardiovascular events in patients with CAD, and both measurement approaches showed similar results [45]. There are also some concerns regarding the use of potentially artificial cut points to construct typology which may result in the likelihood of spurious results [46]. It has also been argued that it would be better to look at the interaction of continuous NA and SI scores to test whether it is indeed the synergistic effect of NA and SI [47].

In this study, when Type D personality was analyzed as a categorical variable, SI has an adverse effect on HbA1c level. It showed that negative affectivity (NA) and social inhibition (SI) are diverse compositions, and they also play different roles in blood glucose control. However, no significant influence on HbA1c was found, when NA × SI term is entered into regression model. These results are consistent with the relationship of Type D personality and coronary heart disease studies [48]. Interestingly, when NA and SI are entered into regression model as continuous variables, effects of NA and NA × SI term on HbA1c level were more significantly. Namely, different analysis methods, where Type D personality was regarded as a categorical or a continuous variable, would produce different results, although the underlying mechanism is still unclear. It further showed that how to deal with the variable of Type D personality deserves further discussion. Relevant studies on this issues should be further conducted in the future in order to explore the underlying mechanism.

Nevertheless, the findings of this study provided new information on the relationship between Type D personality and HbA1c level in patients with T2DM. Being similar with previous study [27], this study also provided the important preliminary evidence regarding the mediating role of medication adherence between Type D personality and HbA1c level. Therefore, the issue regarding personality patterns in patients with diabetes mellitus should be paid high attention; particularly, the medication adherence of patients with Type D personality should be intervened in the clinical management of patients with T2DM.

## 5. Conclusions

To the best of our knowledge, this may be the first follow-up study to explore that Type D personality not only had a direct but also had an indirect influence on HbA1c level through medication adherence. Medication adherence acts as a mediator variable between Type D personality and HbA1c level. More importantly, these findings contribute to a growing body of literature exploring the association between Type D personality and HbA1c level when Type D personality is considered as a continuous variable.

## Figures and Tables

**Table 1:** Sample characteristics.

	Total sample (*n* = 330)	Type D personality (*n* = 92)	Non-Type D personality (*n* = 238)	*P* value
Personal income last year	5.02 ± 9.20	4.28 ± 8.58	5.30 ± 9.44	*t* = 0.901, *P* = 0.368
Reimbursement ratio of treatment costs	77.25 ± 24.43	72.03 ± 31.48	79.27 ± 20.80	*t* = 2.430, *P* = 0.016

**Table 2:** The comparison of the clinical outcomes between the patients with or without Type D personality.

	Total (*n* = 330)	Type D personality (*n* = 92)	Non-Type D personality (*n* = 238)	*P* value
Duration	7.872 ± 5.477	9.228 ± 6.722	7.349 ± 4.827	*t* = −2.82, *P* = 0.005
The number of complications	2.176 ± 1.785	2.467 ± 2.019	2.063 ± 1.676	*t* = −1.85, *P* = 0.065
Cost of treating diabetes	0.842 ± 1.788	1.092 ± 3.244	0.746 ± 0.592	*t* = −1.58, *P* = 0.114
BMI	25.010 ± 3.154	25.668 ± 3.054	24.755 ± 3.162	*t* = −2.37, *P* = 0.018
HbA1c at baseline	7.312 ± 2.004	7.214 ± 1.846	7.350 ± 2.064	*t* = 0.55, *P* = 0.581
HbA1c at T2	7.017 ± 1.003	7.514 ± 1.376	6.824 ± 0.733	*t* = −5.88, *P* < 0.001
Medication adherence	5.44 ± 2.445	4.323 ± 2.505	5.8981 ± 2.258	*t* = 2.257, *P* < 0.001

**Table 3:** OLS and RIF regression analyses predicting HbA1c level at T2.

	Step 1	Step 2	Step 3	Step 1	Step 2	Step 3
	OLS_1	OLS_2	OLS_3	RIF 25th	RIF 50th	RIF 75th
Age	−0.018^*∗∗*^	0.010^*∗∗*^	0.011^*∗∗∗*^	−0.006	0.004	0.020^*∗∗*^
	(0.008)	(0.004)	(0.004)	(0.007)	(0.005)	(0.008)
Gender	0.516^*∗∗∗*^	0.176^*∗∗*^	0.160^*∗*^	−0.078	0.156	0.080
	(0.183)	(0.087)	(0.086)	(0.123)	(0.104)	(0.177)
Education	−0.024	0.095	0.103^*∗*^	−0.158	−0.023	−0.003
	(0.144)	(0.064)	(0.062)	(0.097)	(0.079)	(0.129)
Marital status	−0.081	−0.038	−0.033	0.022	0.111^*∗∗*^	0.252^*∗∗∗*^
	(0.084)	(0.038)	(0.037)	(0.055)	(0.045)	(0.083)
Personal income last year	0.016	−0.005	−0.004	−0.001	−0.005	−0.007
	(0.010)	(0.005)	(0.004)	(0.005)	(0.006)	(0.010)
Reimbursement ratio of treatment costs	−0.012^*∗∗∗*^	−0.004^*∗∗*^	−0.004^*∗∗*^	−0.005^*∗*^	−0.001	−0.002
	(0.004)	(0.002)	(0.002)	(0.002)	(0.002)	(0.004)
Duration		−0.020^*∗∗*^	−0.023^*∗∗∗*^	0.009	−0.025^*∗∗*^	−0.077^*∗∗∗*^
		(0.009)	(0.009)	(0.015)	(0.011)	(0.017)
Complications		0.011	0.010	0.138^*∗∗∗*^	0.071^*∗∗*^	0.161^*∗∗∗*^
		(0.027)	(0.026)	(0.038)	(0.033)	(0.052)
Medical cost of diabetes		0.046^*∗∗*^	0.038^*∗*^	0.037	0.077^*∗*^	0.069^*∗∗*^
		(0.023)	(0.022)	(0.035)	(0.040)	(0.033)
Family history		0.071	0.118	0.506^*∗∗∗*^	0.359^*∗∗∗*^	0.208
		(0.083)	(0.082)	(0.127)	(0.102)	(0.171)
BMI		−0.004	−0.016	0.040^*∗*^	−0.011	0.011
		(0.013)	(0.013)	(0.021)	(0.017)	(0.026)
Baseline HbA1c value		0.771^*∗∗∗*^	0.771^*∗∗∗*^	0.246^*∗∗∗*^	0.292^*∗∗∗*^	0.655^*∗∗∗*^
		(0.021)	(0.020)	(0.026)	(0.026)	(0.042)
Sleep time per day		0.005	0.037	−0.016	0.058^*∗*^	0.239^*∗∗∗*^
		(0.025)	(0.026)	(0.040)	(0.034)	(0.045)
Exercise time per week		0.012	0.013	0.096^*∗∗∗*^	0.027	−0.000
		(0.017)	(0.017)	(0.027)	(0.022)	(0.037)
Drinking alcohol		−0.153	−0.104	−0.310^*∗∗*^	−0.211^*∗*^	0.054
		(0.095)	(0.094)	(0.140)	(0.114)	(0.199)
Type D personality			0.357^*∗∗∗*^	−0.057	−0.073	0.646^*∗∗∗*^
			(0.094)	(0.135)	(0.118)	(0.204)
_cons	8.974^*∗∗∗*^	1.277^*∗∗∗*^	1.147^*∗∗*^	4.146^*∗∗∗*^	4.248^*∗∗∗*^	0.042
	(0.725)	(0.477)	(0.469)	(0.661)	(0.529)	(0.929)

Adjust-*R*^2^	0.092	0.837	0.844	0.298	0.372	0.530
*P*	0.000	0.000	0.000	0.000	0.000	0.000

Note: standard errors in parentheses; ^*∗*^*P* < 0.1, ^*∗∗*^*P* < 0.05, and ^*∗∗∗*^*P* < 0.01.

**Table 4:** Type D personality components: NA and SI on higher HbA1c level at T2.

	Step 1	Step 2	Step 3	Step 4
	OLS_NA SI	OLS_NA SI	OLS_NA SI	OLS_NA SI
Age	0.011^*∗∗∗*^	0.011^*∗∗∗*^	0.011^*∗∗*^	0.011^*∗∗*^
	(0.004)	(0.004)	(0.004)	(0.004)
Gender	0.146^*∗*^	0.145^*∗*^	0.159^*∗*^	0.169^*∗*^
	(0.087)	(0.087)	(0.087)	(0.086)
Education	0.100	0.101	0.119^*∗*^	0.116^*∗*^
	(0.062)	(0.062)	(0.064)	(0.063)
Marital status	−0.036	−0.031	−0.043	−0.036
	(0.037)	(0.038)	(0.038)	(0.037)
Personal income last year	−0.004	−0.004	−0.004	−0.005
	(0.004)	(0.004)	(0.004)	(0.004)
Reimbursement ratio of treatment costs	−0.004^*∗∗*^	−0.003^*∗*^	−0.004^*∗∗*^	−0.004^*∗∗*^
	(0.002)	(0.002)	(0.002)	(0.002)
Duration	−0.023^*∗∗*^	−0.023^*∗∗∗*^	−0.023^*∗∗*^	−0.024^*∗∗∗*^
	(0.009)	(0.009)	(0.009)	(0.009)
Complications	0.009	0.008	0.009	0.005
	(0.026)	(0.026)	(0.026)	(0.026)
Cost of treating diabetes	0.039^*∗*^	0.039^*∗*^	0.042^*∗*^	0.047^*∗∗*^
	(0.022)	(0.023)	(0.023)	(0.023)
Family history	0.096	0.102	0.094	0.109
	(0.084)	(0.084)	(0.084)	(0.084)
BMI	−0.014	−0.015	−0.012	−0.016
	(0.013)	(0.013)	(0.013)	(0.013)
Baseline HbA1c value	0.776^*∗∗∗*^	0.778^*∗∗∗*^	0.767^*∗∗∗*^	0.775^*∗∗∗*^
	(0.022)	(0.022)	(0.021)	(0.021)
Sleep time every day	0.028	0.027	0.021	0.017
	(0.030)	(0.030)	(0.032)	(0.032)
The weekly exercise time	0.012	0.011	0.013	0.009
	(0.017)	(0.017)	(0.017)	(0.017)
Drinking behavior	−0.105	−0.102	−0.098	−0.098
	(0.094)	(0.094)	(0.095)	(0.094)
NA Type	−0.076	−0.106		
	(0.131)	(0.139)		
SI Type	0.395^*∗∗∗*^	0.187		
	(0.120)	(0.349)		
NA Type × SI Type		0.242		
		(0.380)		
NA score			−0.014	−0.031^*∗∗*^
			(0.013)	(0.015)
SI score			0.034^*∗∗∗*^	−0.032
			(0.012)	(0.032)
NA score × SI score				0.004^*∗∗*^
				(0.002)
_cons	1.167^*∗∗*^	1.179^*∗∗*^	1.275^*∗∗∗*^	1.467^*∗∗∗*^
	(0.472)	(0.472)	(0.484)	(0.489)

Adjust-*R*^2^	0.844	0.843	0.842	0.843
*P*	0.000	0.000	0.000	0.000

Note: standard errors in parentheses; ^*∗*^*P* < 0.1, ^*∗∗*^*P* < 0.05, and ^*∗∗∗*^*P* < 0.01.

In step 1 and step 2, Type D personality was analyzed as a categorical variable; in step 3 and step 4, NA and SI were analyzed as the continuous variables.

**Table 5:** Mediation effect between Type D personality and HbA1c.

	A path	B path	C path	C′ path
	Direct effects of Type D personality on medication adherence	Direct effects of medication adherence on HbA1c	Direct effects of Type D personality on HbA1c	Coeffect of Type D personality and medication compliance
Type D personality	−1.903^*∗∗∗*^		0.357^*∗∗∗*^	0.247^*∗∗*^
	(0.235)		(0.094)	(0.102)
Medication adherence		−0.080^*∗∗∗*^		−0.058^*∗∗*^
		(0.021)		(0.022)
_cons	10.704^*∗∗∗*^	2.082^*∗∗∗*^	1.147^*∗∗*^	1.766^*∗∗∗*^
	(1.174)	(0.510)	(0.469)	(0.523)

Adjust-*R*^2^	0.519	0.844	0.844	0.847
*P*	0.000	0.000	0.000	0.000
	Mediation effect	42.64%	Mediating effect of total effect	54.43%

Note: (1) standard errors in parentheses; ^*∗*^*P* < 0.1, ^*∗∗*^*P* < 0.05, and ^*∗∗∗*^*P* < 0.01.

(2) Mediation effect calculation: *E* = *β*(A  path)*∗β*(B  path)/*β*(C  path) = (−1.903)*∗*(−0.080)/0.357 = 42.64%.

(3) Mediating effect of total effect calculation: *P* = *E*(Mediation  Effect)/(*E*(Mediation  Effect) + *β*(C  path)) = 42.64%/(42.64% + 37.7%) = 54.43%.

## Abbreviations

T2DM: Type 2 diabetes mellitus
FPG: Fasting plasma glucose
RPG: Random plasma glucose
OGTT: Oral glucose tolerance test
HbA1c: Glycated haemoglobin
OLS: Ordinary Least Square Regression
RIF: Recentered Influence Function Regression
BMI: Body mass index
WHO: World Health Organization
IDF: International Diabetes Federation
ADA: American Diabetes Association
NA: Negative affectivity
SI: Social inhibition
MMAS-8-CN: Chinese version of the Medication Adherence Scale.

## Additional Points

*Limitations.* There are some limitations of this study which should be acknowledged. First, this study was limited due to the small sample size and we only adjusted some potential confounders, which might have an impact on HbA1c level in the multiple regression models. Other potential confounders might be ignored. In future studies, larger sample should be included to analyze the influence mechanism between Type D personality and HbA1c. Second, this study did not put early diabetes record variables into the regression model; therefore it may result in some bias. Future studies should consider patient's condition in the past and improve the survey design. Third, the correlational study design precluded casual inferences, and intervention studies were required to establish whether modifying medication adherence can help in controlling HbA1c.
